# Mixed‐Dimensional Formamidinium Bismuth Iodides Featuring In‐Situ Formed Type‐I Band Structure for Convolution Neural Networks

**DOI:** 10.1002/advs.202200168

**Published:** 2022-03-20

**Authors:** June‐Mo Yang, Ju‐Hee Lee, Young‐Kwang Jung, So‐Yeon Kim, Jeong‐Hoon Kim, Seul‐Gi Kim, Jeong‐Hyeon Kim, Seunghwan Seo, Dong‐Am Park, Jin‐Wook Lee, Aron Walsh, Jin‐Hong Park, Nam‐Gyu Park

**Affiliations:** ^1^ School of Chemical Engineering Energy Frontier Laboratory Sungkyunkwan University Suwon 16419 Korea; ^2^ Department of Electrical and Computer Engineering Sungkyunkwan University Suwon 16419 Korea; ^3^ Department of Materials Science and Engineering Yonsei University Seoul 03722 Korea; ^4^ Sungkyunkwan Advanced Institute of Nanotechnology (SAINT) Sungkyunkwan University Suwon 16419 Korea; ^5^ Department of Materials Imperial College London London SW7 2AZ UK

**Keywords:** artificial synapses, convolution neural networks, energy consumption, formamidinium bismuth iodide, memristors, mixed‐dimensional, type I band alignment

## Abstract

For valence change memory (VCM)‐type synapses, a large number of vacancies help to achieve very linearly changed dynamic range, and also, the low activation energy of vacancies enables low‐voltage operation. However, a large number of vacancies increases the current of artificial synapses by acting like dopants, which aggravates low‐energy operation and device scalability. Here, mixed‐dimensional formamidinium bismuth iodides featuring in‐situ formed type‐I band structure are reported for the VCM‐type synapse. As compared to the pure 2D and 0D phases, the mixed phase increases defect density, which induces a better dynamic range and higher linearity. In addition, the mixed phase decreases conductivity for non‐paths despite a large number of defects providing lots of conducting paths. Thus, the mixed phase‐based memristor devices exhibit excellent potentiation/depression characteristics with asymmetricity of 3.15, 500 conductance states, a dynamic range of 15, pico ampere‐scale current level, and energy consumption per spike of 61.08 aJ. A convolutional neural network (CNN) simulation with the Canadian Institute for Advanced Research‐10 (CIFAR‐10) dataset is also performed, confirming a maximum recognition rate of approximately 87%. This study is expected to lay the groundwork for future research on organic bismuth halide‐based memristor synapses usable for a neuromorphic computing system.

## Graphical abstract

Mixed‐dimensional formamidinium bismuth iodides exhibit highly linear potentiation/depression characteristics with energy consumption as low as 61.08 aJ for the convolutional neural network (CNN) due to a large number of defects and in‐situ formed type I band alignment.

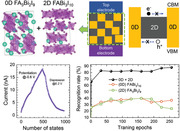

## 1 Introduction

As the amount of video, images, and other unstructured data increases exponentially, neuromorphic computing technique was suggested to process such data more efficiently, compared to serial computing based on von‐Neumann architecture.^[^ ^1^, ^2^ ^]^ Since neuromorphic computing conceptually originated from the operation of the biological human brain, in which neurons and synapses are connected in parallel, it is convenient to implement parallel computing with high‐speed and energy‐efficient data processing.^[^ ^3^, ^4^ ^]^ In recent years, to realize the architecture and functionality for neuromorphic computing, several kinds of two‐terminal memory devices including phase‐change memory (PCM), magnetoresistive random‐access memory (MRAM) and memristor (a portmanteau of memory resistor) are suggested.^[^ ^5^, ^6^, ^7^ ^]^ Among them, memristor devices are highly suitable for neuromorphic computing because scalability, switching speed and endurance are better than those of other devices and the operating mechanism is similar to that of biological synapses.^[^ ^8^, ^9^ ^]^ The memristor devices can be divided into two types according to resistive switching mechanisms: electrochemical metallization memory (ECM) and valence change memory (VCM).^[^ ^10^ ^]^ In the ECM device, resistive switching occurs by the formation and rupture of conducting filament via oxidation and reduction of metal.^[^ ^10^ ^]^ On the other hand, in the VCM devices, migration of defect leads to forming conducting filament or changing Schottky barrier at the interface between switching layer and metal electrode.^[^ ^9^, ^10^ ^]^ ECM is generally not favorable to energy consumption because the operating voltage is highly dependent on the active metals such as Ag, Cu, Ni, etc.^[^ ^11^, ^12^ ^]^ Unlike ECM devices, the VCM devices using media such as metal oxides can feature very low operating voltages, which can be further reduced to less than 1 V by generating plenty of vacancies in the metal oxide.^[^ ^13^ ^]^ Furthermore, vacancies are beneficial to achieving linear P/D characteristics.^[^ ^13^ ^]^ However, a large number of vacancies can increase the overall current of artificial synapses because charge carriers are also generated in the case that vacancies act like dopants,^[^ ^14^, ^15^ ^]^ which is a disadvantage to energy consumption. In addition, high level of current may disturb scalable neuromorphic computing because the amount of current that flows in a wire is limited and the raised current can be saturated in artificial neurons connected with artificial synapses.^[^ ^16^, ^17^ ^]^ Therefore, new materials for switching medium of artificial synapses are required to realize synaptic characteristics in terms of recognition accuracy, operating current, and energy efficiency. Recently, halide perovskite materials have been considered toward the memristor‐based synapses with a low operating voltage because of low activation energy for ion migration less than 1 eV.^[^ ^18^, ^19^, ^20^, ^21^ ^]^ Artificial synapses with halide perovskite materials exhibited relatively low energy consumption and successfully imitated biological synapse behaviors such as P/D and spike‐timing‐dependent plasticity (STDP) characteristics.^[^ ^8^, ^9^, ^21^ ^]^ However, lead halide perovskite materials have been also suffered from behavior of vacancies acting like dopant and exhibited several problems such as small dynamic range, linearity and micro‐ampere scale current.^[^ ^21^, ^22^, ^23^, ^24^ ^]^

Here, we report a mixed‐dimensional formamidinium bismuth iodides consisting of two‐dimensional (2D) FABi_3_I_10_ (FA = formamidinium) and zero‐dimensional (0D) FA_3_Bi_2_I_9_ as a switching medium of artificial synapse with low energy consumption. Low crystallinity and type‐I band structure, which are achieved by combining 2D and 0D phases, induce linear P/D characteristic with considerable dynamic range and reduce the current level down to pico ampere scale while maintaining the current controllability. The crystal structure and film morphology are analyzed using X‐ray diffraction (XRD) and scanning electron microscope (SEM). We then conduct space charge limited current (SCLC) and capacitance measurements for estimating defect density and conductivity, and the temperature‐dependent electrical measurement for calculating the activation energy for ion migration. Type‐I band alignment in the mixed phase is confirmed by density functional theory (DFT) calculation. In an aspect of synaptic device operation, the exhibitory postsynaptic current (EPSC), P/D, spike‐number‐dependent plasticity (SNDP), spike‐voltage‐dependent plasticity (SVDP) and STDP characteristics are discussed, and especially, we present asymmetricity, dynamic range, and energy consumption extracted from the P/D characteristic curve. Finally, we demonstrate the applicability of our artificial synapse for convolutional neural networks via training and inference tasks for the CIFAR‐10 dataset using the “DNN+ NeuroSim” simulator^[^ ^25^ ^]^ and the modified National Institute of Standards and Technology (MNIST) dataset using the “NeuroSim+ MLP” simulator.^[^ ^26^ ^]^

## 2 Results and Discussion

**Figure 1:**
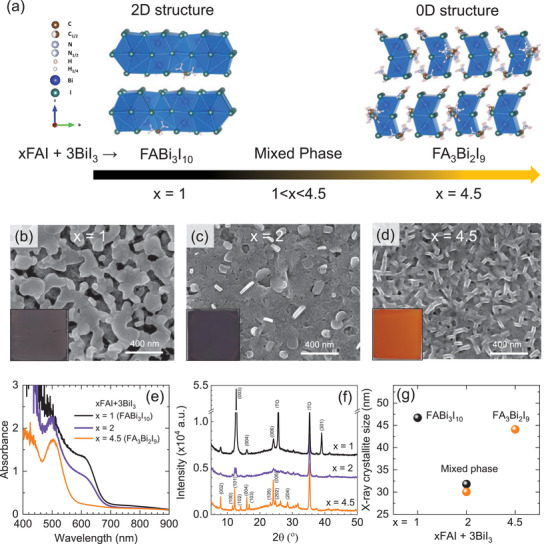
Phase and morphology evolution depending on FAI concentration in *x*FAI + 3BiI_3_. a) Schematic illustration of crystal structure of formamidinium bismuth iodides depending on composition of precursor solution. Plane view of SEM images of b) 2D FABi_3_I_10_ formed by *x* = 1, c) a mixed phase formed by *x* = 2 and d) 0D FA_3_Bi_2_I_9_ produced by *x* = 4.5. e) Absorbance of materials formed by *x* = 1 (FABi_3_I_10_), 2 (mixed phase) and 4.5 (FA_3_Bi_2_I_9_) in *x*FAI + 3BiI_3_. Films were deposited on glass substrate. f) X‐ray diffraction (XRD) patterns and g) X‐ray crystallite size of materials formed by *x* = 1, 2 and 4.5 in *x*FAI + 3BiI_3_. Films were deposited on ITO substrate.

Crystal structure of the spin‐coated formamidinium bismuth iodide is found to depend on the molar ratio of FAI to BiI_3_ in the precursor solution. 2D layered structure (FABi_3_I_10_) is formed by FAI: BiI_3_ = 1: 3, while a 0D hexagonal structure with the chemical formula of FA_3_Bi_2_I_9_ is obtained by FAI: BiI_3_ = 4.5: 3.^[^ ^27^, ^28^, ^29^ ^]^ The 2D–0D mixed phase can be formed when *x* is between 1 and 4.5 in *x*FAI: BiI_3_. In **Figure** **1**a, the crystal structure of 2D and 0D formamidinium bismuth iodide is displayed along with the reaction product depending on the molar ratio of precursors. The top‐view SEM images in Figure 1b–d show that large grains with a porous nature are observed for 2D FABi_3_I_10_ (Figure 1b), whereas rod‐shaped grains are connected for 0D FA_3_Bi_2_I_9_ (Figure 1d). The film formed by FAI: BiI_3_ = 2: 3 exhibits that a bright rod is lodged in the dark island in Figure 1c, which is indicative of morphological mixture of FABi_3_I_10_ and FA_3_Bi_2_I_9_. Thickness of the 2D FABi_3_I_10_ film, the 2D–0D mixed phase and the 0D FA_3_Bi_2_I_9_ film is estimated to be ≈279 nm, ≈310 nm and ≈393 nm, respectively, as measured by cross‐sectional SEM (Figure S1, Supporting Information). Color of films is changed by the molar ratio of FAI and BiI_3_. FABi_3_I_10_ and the 2D‐0D mixed phase show brown‐purple gray, while FA_3_Bi_2_I_3_ turns orange. This phenomenon is indicative of a change in the band gap. Band gap of FABi_3_I_10_ and FA_3_Bi_2_I_9_ is estimated to be 1.83 eV and 2.25 eV, respectively, according to Tauc plots (Figure S2, Supporting Information) based on UV–visible absorbance (Figure 1e). Two absorption bands are observed for the 2D–0D mixed phase in Figure 1e, which is evidence of the mixture of 2D and 0D with band gap of 1.86 eV and 2.21 eV. In addition, XRD patterns in Figure 1f show clearly the co‐existence of 2D and 0D in the reaction product of FAI: BiI_3_ = 2: 3, while FABi_3_I_10_ and FA_3_Bi_3_I_9_ are well indexed to 2D layer structure and 0D hexagonal structure.^[^ ^27^, ^28^, ^29^ ^]^ It is noted that peak intensity is significantly lower for the 2D‐0D mixed phase than for pure 2D and 0D phase, which indicates that crystallinity is reduced in the mixed phase. Full width half maximum (FWHM) of the (003) peak at 12.72° for the FABi_3_I_10_ film is 0.17 and that of the (101) peak at 12.30° for FA_3_Bi_2_I_9_ films is 0.18, while FWHMs are increased to 0.25 for the (003) peak and 0.26 for the (101) peak in the 2D–0D mixed phase. This indicates that crystallite size, estimated by Scherrer equation,^[^ ^30^ ^]^ is decreased from ≈45 to ≈30 nm upon mixing 2D and 0D phase (Figure 1g). In the 2D–0D mixed phase formed by the ratio of FAI: BiI_3_ = 2: 3, relatively small crystallite size with low crystallinity is probably due to inhomogeneous crystallization of different phases.^[^ ^31^, ^32^ ^]^

**Figure 2:**
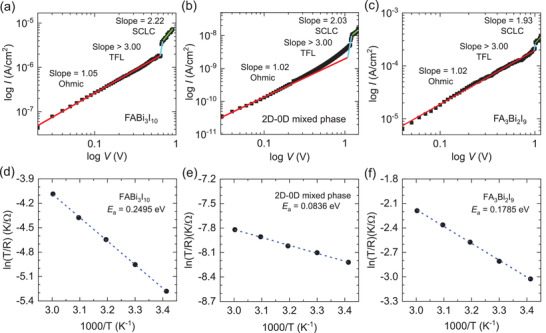
SCLC and activation energy for migration of iodide vacancies. Dark current (*I*)–Voltage (*V*) curves of a) FABi_3_I_10_, b) 2D‐0D mixed phase and c) FA_3_Bi_2_I_9_. Au/formamidinium bismuth iodides/ITO devices were used, from which *V*_TFL_ was estimated to be 0.64 V, 1.17 V and 0.98 V for FABi_3_I_10_, the mixed phase and FA_3_Bi_2_I_9_, respectively. Ln(T/R) as a function of inverse temperature for d) FABi_3_I_10_, e) 2D‐0D mixed phase and f) FA_3_Bi_2_I_9_.

SCLC in **Figure** **2**a–c, activation energy in Figure 2d–f, and capacitance in Figure S3, Supporting Information, are measured to understand defect migration and properties of charge carriers. Defect density (*N*_defect_) can be calculated based on trap‐filled limit voltage (*V*_TFL_) from SCLC data using Equation 1,^[^ ^33^ ^]^

(1) $$N_{\mathrm{defect}}=\frac{2\varepsilon \varepsilon_{0}V_{TFL}}{eL^{2}}$$

where *e* is the elementary charge and *L* is the film thickness. Permittivity *εε_0_* (*ε_0_* is the vacuum permittivity and *ε* is the dielectric constant) is related to capacitance (*C*) in Equation 2, where *A*_1_ stands for the device area for capacitance measurement:

(2) $$C=\varepsilon \varepsilon_{0}\frac{A_{1}}{L}$$

By combining Equation 1 and 2, *N*_defect_ is calculated from Equation 3.

(3) $$N_{\mathrm{defect}}=\frac{2CV_{TFL}}{eA_{1}L}$$

*N*_defect_ is estimated to be 2.38 × 10^15^ cm^−3^, 3.46 × 10^15^ cm^−3^ and 1.55 × 10^16^ cm^−3^ for FABi_3_I_10_, the 2D‐0D mixed phase and FA_3_Bi_2_I_9_, respectively (see Table S1, Supporting Information). Higher *N*_defect_ for the mixed phase is presumably due to low crystallinity with smaller crystallite size. Since defect is considered to originate from iodide vacancy,^[^ ^34^, ^35^, ^36^ ^]^ iodide vacancy might be moved to the electrode interface when voltage is applied. Activation energy (*E*_a_) for vacancy migration is thus important, which is estimated by using Nernst‐Einstein relation in Equation 4,

(4) $$\sigma \left(T\right)=\frac{\sigma_{0}}{T}e^{-E_{a}/kT}$$

where *σ*(*T*) and *k* are the temperature‐dependent conductivity and the Boltzmann constant, respectively. *E*_a_ is determined from the ln*σ ‐*‐ (1/*T*) plot in Figure 2d–f, where the mixed phase shows a lower *E*_a_ of 0.0836 eV than 0.2495 eV for FABi_3_I_10_ and 0.1785 eV for FA_3_Bi_2_I_9_. This indicates that the mixed phase is beneficial to the migration of iodide vacancy.

Characteristics of charge carrier are investigated using Mott‐Gurney's square law in Equation 5,^[^ ^33^ ^]^

(5) $$\frac{I}{A_{2}}=\;\frac{9\varepsilon \varepsilon_{0}\mu V^{2}}{8L^{3}}$$

where *I*, *A*_2_, *μ* and *V* are the dark current density, the active area for SCLC, the carrier mobility and the applied voltage, respectively. By combining Equations 5 and 2, *μ* can be estimated by Equation 6.

(6) $$\mu =\frac{8L^{2}A_{1}}{9CA_{2}}\;\frac{I}{V^{2}}$$

Based on the data listed in Table S1, Supporting Information, *μ* is determined to be 2.18 × 10^−2^ cm^2^ V^− 1^s^−1^ for FABi_3_I_10_, 5.47 × 10^−7^ cm^2^ V^− 1^s^−1^ for the 2D–0D mixed phase and 9.19 × 10^−2^ cm^2^ V^− 1^s^−1^ for FA_3_Bi_2_I_9_ (see Table S2, Supporting Information). Carrier density (*n*) is also evaluated from Equation 7 ^[^ ^37^ ^]^ using the current of the ohmic region in SCLC,

(7) $$I=neA_{1}v_{d}$$

where *v*_d_ is the drift velocity of charge carriers and has relation with *μ* in Equation 8.^[^ ^38^ ^]^

(8) $$v_{d}=\mu \frac{V}{L}$$

Equation 9 can be established by combining Equations 7 and 8,

(9) $$n=\frac{L}{\mu eA_{1}}\;\frac{I}{V}$$

where *I* and *V* in ohmic region should be used. Based on the estimated *n* in Table S2 (*n* = 6.82 × 10^14^ cm^−3^ for FABi_3_I_10_, 1.99 × 10^15^ cm^−3^ for the 2D‐0D mixed phase and 1.50 × 10^15^ cm^−3^ for FA_3_Bi_2_I_9_), conductivity (*δ*) is obtained by Equation 10.^[^ ^39^ ^]^

(10) δ=enμ

As compared to *δ* of FABi_3_I_10_ (2.38 × 10^−7^ Scm^−1^) and FA_3_Bi_2_I_9_ (2.21 × 10^−5^ Scm^−1^), the 2D‐0D mixed phase is found to demonstrate a significantly reduced *δ* of 1.75 × 10^−10^ Scm^−1^. Despite the increased *n* due to higher *N*_defect_,^[^ ^22^, ^23^ ^]^ the decreased *δ* is mainly attributed to the five orders lower *μ* for the 2D–0D mixed phase than for the pure 2D and 0D phase.

**Figure 3:**
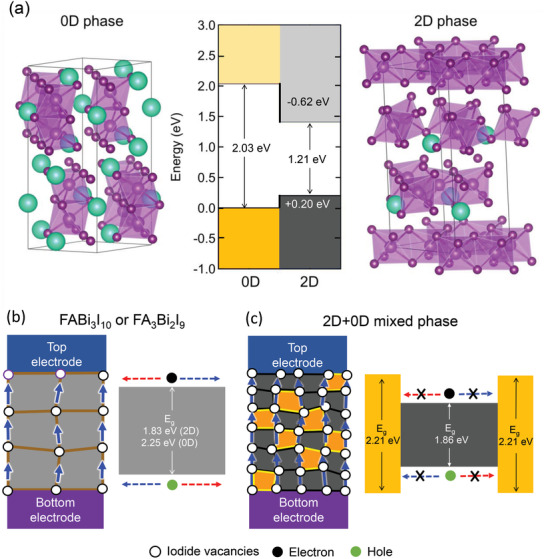
Characteristic related to energy state and ion migration. a) Crystal structures of 0D and 2D phases were used for DFT calculations and the resulting electronic band offset between the two phases (conduction band offset = 0.62 eV and valence band offset = 0.20 eV). Band structure and properties of ion migration for b) pure materials and c) the mixed phase.

In order to understand the unusual carrier property of low conductivity yet high mobility for the 2D–0D mixed phase, computational simulation is performed. When two different semiconductors form a junction, movement of charge carriers through the junction is mainly affected by the type of band offset between the two materials.^[^ ^40^, ^41^ ^]^ To identify the type of band offset between the 0D phase and the 2D phase, we have performed first‐principles density functional theory (DFT) calculations (detailed description is shown in the Methods section in the supporting information). Our calculation based on interface super lattice model between the 0D phase and the 2D phase indicates that formation of type I band offset is possible where electrons and holes can be easily trapped at low band gap 2D phase (**Figure** **3**a), which is well corresponded to absorbance results of the mixed phase in Figure S2, Supporting Information. Such behavior has been observed for other metal halide heterojunction systems including perovskite/PbS and 3D/2D perovskite heterojunctions.^[^ ^42^, ^43^, ^44^ ^]^ Formation of type I heterojunction in the mixed phase can disturb conduction of charge carriers and thereby induce large capacitance.^[^ ^33^, ^42^, ^43^, ^44^ ^]^ Based on our analysis, carrier transport and ion migration under electric field are schematically illustrated in memristor devices based on pure 2D or 0D material (Figure 3b) and the 2D–0D mixed material (Figure 3c). For FABi_3_I_10_ and FA_3_Bi_2_I_9_ in Figure 3b, *μ* is expected to be relatively high because of no energy barrier that prevents carrier transport despite the difficulty in migration of iodide vacancies due to relatively low carrier density induced by low defect density in high crystallinity. This can explain high conductivity together with high activation energy for the migration of iodide vacancies. On the other hand, carrier transport in the 2D–0D mixed phase is expected to be seriously disturbed due to type I band alignment (Figure 3c), which can explain the low *μ* and thereby low *δ*. Despite low *μ*, migration of iodide vacancies seems to be easier thanks to the high density of carriers and low *E*_a_. From the viewpoint of neuromorphic computing, it is expected that low *δ* is beneficial to energy consumption and scalability. Furthermore, a large number of defects and low *E*_a_ for iodide vacancies is also beneficial to linearity and dynamic range.^[^ ^13^, ^16^ ^]^

**Figure 4:**
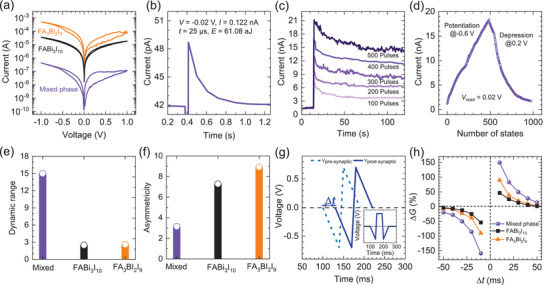
Memristive characteristics and synaptic behavior. a) *I‐*‐*V* characteristics of the memristor devices for FABi_3_I_10_, FA_3_Bi_2_I_9_ and the mixed phase. b) EPSC properties observed at a 25 µs pulse of ‐0.02 V, where cell size was 1963 µm^2^. A full current‐time profile is presented in Figure S7, Supporting Information. c) Long‐term potentiation is dependent on the number of pulses. d) Potentiation and depression for the mixed phase based memristor devices depending on the number of pulses, where 500 consecutive negative pulses (−0.6 V, 300 µs) for potentiation were followed by 500 positive pulses (0.2 V, 300 µs) for depression. 0.02 V reading voltage was applied after each negative and positive pulse. e) Dynamic range and f) asymmetricity of the memristor employing FABi_3_I_10_, the mixed phase and FA_3_Bi_2_I_9_. g) Pre‐synaptic and post‐synaptic spikes for emulating STDP. h) STDP behavior the memristor employing FABi_3_I_10_, the mixed phase and FA_3_Bi_2_I_9_.

*I*–*V* characteristic of memristor devices is displayed in **Figure** **4**a, where positive voltage sweep (0 V → +1 V → 0 V) is followed by negative voltage sweep (0 V → −1 V → 0 V). ON/OFF ratio (low resistance state/high resistance state) for the mixed phase is determined to be 6.75 which is higher than those for FABi_3_I_10_ (1.25) and FA_3_Bi_2_I_9_ (2.25). Moreover, when negative voltage sweeps from 0 V to −1 V and then back to 0 V, defined as one cycle, are repeated for five cycles in our memristor devices (Figure S4, Supporting Information). The current of the mixed phase at −1 V is increased by more than 4 times, while the increased current for FABi_3_I_10_ and FA_3_Bi_2_I_9_ is less than 2 times during 5 cycles. Different ON/OFF ratio and *I*–*V* behavior at negative voltage sweeps might be related to *N*_defect_ and *E*_a_, where the smaller the *N*_defect_ and/or the higher *E*_a_, the lower the ON/OFF ratio, and vice versa. Thus, large *N*_defect_ and low *E*_a_ for the mixed phase seem to be responsible for the relatively high ON/OFF ratio. It is noted that overall current is lower for the mixed phase than for pure FABi_3_I_10_ and FA_3_Bi_2_I_9_, which is reproduced by repetitive resistive switching (Figure S5, Supporting Information). The basis for the low current is related to the type I band structure of the mixed phase because transport of charge carriers is disturbed despite enhanced density of charge carriers. The morphology of films can be one of the factor affecting the electric current of memristor devices. It was reported, however, that similar resistive switching behavior was observed despite a large change in morphology of the halide perovskite active layer, whereas, energy state can affect resistive switching property.^[^ ^45^, ^46^, ^47^, ^48^ ^]^ Thus, the morphology of formamidinium bismuth iodides is expected to hardly affect the switching behavior of memristor devices. Resistive switching type is important to understand the switching mechanism. From the active‐area dependent current measurements in Figures S6 and S7, Supporting Information,^[^ ^39^ ^]^ the formamidinium bismuth iodide based memristor is working like interface‐type since the current is proportional to the active area (the current is increased by more than 20 times when the active area is increased by about 16 times from 1963 to 31415 µm^2^.^[^ ^49^ ^]^

We then investigate synaptic behavior of the memristor devices (Figure 4), where the cell area is 7856 µm^2^ except for Figure 4b. When a 25 µs pulse of −0.02 V is applied to a memristor device employing the mixed phase, current is increased from 0.520 nA to 0.603 nA and then decayed to 0.523 nA. This results in the change in synaptic weight (ΔG) of +0.50% as calculated by (*I*_final_ − *I*_initial_)/*I*_initial_ (Figure S8, Supporting Information).^[^ ^8^, ^9^ ^]^ This change corresponds to EPSC of biological synapses due to a partial migration of iodide vacancies in the switching layer from the mixed phase to the bottom PEDOT:PSS/ITO electrode (PEDOT:PSS = Poly(3,4‐ethylenedioxythiophene):poly(styrenesulfonate)). Energy consumption for EPSC is estimated to be 0.585 fJ from a peak current of 1.169 nA under a 25 µs pulse at −0.02 V (Energy consumption = |−0.02 V| × 1.169 nA × 25 µs = 0.5845 × 10^−15^ J ( = 0.585 fJ)). Energy consumption of 0.585 fJ is the lowest among the reported data based on halide perovskite materials (**Table** **1**). Furthermore, energy consumption of 61.08 aJ (|−0.02 V| × 122 pA × 25 µs) for EPSC is realized by downsizing the cell area to 1963 µm^2^ (Figure 4b and Figure S9, Supporting Information) that is closed to the size of the tip in our measuring instrument. We also confirm that ΔG is raised from + 0.50% to + 0.74%, + 0.92%, + 1.10% and +1.25% when the number of pulses applied is increased to 2, 3, 4 and 5. (Figure S10a,b and Table S3, Supporting Information). It indicates that repetitive pulses of −0.02 V can increase the amount of iodide vacancies migrated to the interface. This phenomenon is defined as spike number dependent plasticity (SNDP). SNDP is similar to an increase of plasticity in biological synapses when small stimulus are repeated.^[^ ^9^, ^48^ ^]^ Moreover, ΔG is also raised from + 0.50% to + 1.25%, + 2.15%, + 2.90% and +3.63% when the absolute voltage values of pulses are increased to −0.03, −0.04, –0.05 and −0.06 V (Figure S11a,b and Table S4, Supporting Information), which indicates that strong pulses can increase the amount of iodide vacancies at the interface, which is defined as spike voltage dependent plasticity (SVDP).^[^ ^9^, ^48^ ^]^ SVDP is similar to the change of plasticity in biological synapses when the strength of stimulation is changed. We also confirm that SNDP and SVDP occurs when cell size is decreased to 1963 µm^2^, where ΔG is raised from + 0.50% to + 0.74%, + 0.92%, + 1.10% and +1.25% when the number of pulses applied is increased from 1 to 5 and ΔG is also raised from + 0.50% to + 1.25%, + 2.15%, + 2.90% (Figure S10c,d and Table S3, Supporting Information) and +3.63% when the absolute voltage value of pulses are increased to −0.03, −0.04, −0.05 and −0.06 V (Figure S11c,d and Table S4, Supporting Information). This short‐term plasticity shows that our memristor device can be used for reservoir computing as well as neuromorphic computing which is discussed at the end of this paper.^[^ ^50^, ^51^ ^]^Energy consumption of SNDP for 2nd, 3rd, 4th and 5th pulses is estimated to be 0.602, 0.626, 0.652 and 0.679 fJ, respectively, and energy consumption of SVDP for −0.03 V, −0.04 V, −0.05 V and −0.06 V pulses is calculated to be 0.877 fJ, 1.163 fJ, 1.510 fJ and 1.765 fJ respectively. Moreover, the energy consumption of the 5th pulse for SNDP is decreased from 0.68 fJ to 0.067 fJ and that of the −0.06 V pulse for SVDP is decreased from 1.76 fJ to 0.19 fJ when the active area is decreased from 7853 µm^2^ to 1963 µm^2^ (Table S3 and Table S4, Supporting Information). These results indicate that not only EPSC but also various synaptic emulation can occur in the 2D‐0D mixed phase with extremely low energy consumption. Moreover, synaptic weight for the mixed phase is well retained for over 100 s when 100, 200, 300, 400 and 500 pulses of −0.6 V is applied and reading voltage is 0.02 V (Figure 4c), which corresponds to long‐term potentiation (LTP) of biological synapses.^[^ ^9^, ^52^ ^]^ *N*_defect_ and *E*_a_ have an influence on not only energy consumption but also P/D. To investigate P/D characteristics, the identical pulses of −0.6 V are applied for potentiation and +0.2 V for depression, where reading voltage is 0.02 V. As shown in Figure 4d, the mixed phase based memristor devices show highly linear P/D characteristics with 500 states. Dynamic range (maximum signal/minimum signal) and asymmetricity are estimated to be 15 (Figure 4e) and of 3.15 (Figure 4f) for the mixed phase, while FABi_3_I_10_ and FA_3_Bi_2_I_9_ based memristor devices show non‐linear P/D characteristics (Figure S12, Supporting Information) resulting in dynamic range as low as ≈2.5 (Figure 4e) and high asymmetricity more than 7 (Figure 4f). Asymmetricity is different from non‐linearity (*β*) of potentiation and depression,^[^ ^53^, ^54^ ^]^ where the method for extracting the *β* is well explained in Figure S13, Supporting Information. High dynamic range with low asymmetricity observed from the mixed phase is highly suitable for pattern recognition based on CNNs or artificial neural networks (ANNs).^[^ ^25^, ^26^ ^]^ In addition to P/D characteristics, emulating STDP related to Hebbian learning rule is an important requirement in artificial synapse,^[^ ^9^, ^55^ ^]^ where STDP indicates that time interval of the spikes between pre‐ and post‐synaptic terminal determines synaptic weight in biological synapses. To mimic STDP, the shapes of two spikes are required to be the same with specific time intervals as shown in Figure 4g, where one spike is applied to pre‐synaptic terminal and the other is applied to post‐synaptic terminal.^[^ ^9^, ^55^ ^]^ Time interval is defined to Δ*t* (Δ*t* = *t*_pre_ − *t*_post_).^[^ ^9^, ^55^ ^]^ Two spikes are converted into net spikes to realize STDP in memristor devices as shown in the inset in Figure 4g ^[^ ^56^, ^57^ ^]^ and various shapes of input spikes for emulating the STDP are demonstrated in Figure S14, Supporting Information.^[^ ^58^ ^]^ If the pre‐synaptic spike arrives after the postsynaptic spike (Δ*t* > 0), the polarity of net spikes is negative and synaptic weight becomes potentiated (Δ*G* > 0).^[^ ^8^, ^9^ ^]^ On the other hand, when the pre‐synaptic spike arrives later than the postsynaptic spike, the polarity of net spikes will be positive, leading to a depressed synaptic weight (Δ*G* < 0).^[^ ^8^, ^9^ ^]^ Consequently, Δ*G* is negatively dependent on the time interval between pre‐synaptic and post‐synaptic spikes, which means the absolute value of synaptic weight is increased as Δ*t* become closer to 0.^[^ ^8^, ^9^ ^]^ Δ*G* for the mixed phase is larger than that for pure 2D and 0D phases (Figure 4h), which means that the mixed phase based memristor devices are more sensitive to the electric stimulation. The results shown in Figure 4 indicates that the mixed phase is highly suitable for artificial synapses in terms of current, energy consumption, P/D characteristic and STDP.

**Table 1:** Comparison of energy consumption of the mixed phase‐based synaptic devices with the reported halide perovskite material‐based synaptic devices

Materials	*E*_a_ for migration of vacancies	Required pulse for synaptic event [Voltage, pulse period]	Energy consumption per synaptic event [fJ]	Ref.
MAPbI_3_	0.360 eV	−11 V, 100 µs	3 × 10^14^	^[^ ^59^ ^]^
MAPbBr_3_	0.058 eV	0.02 V, 100 ms	20	^[^ ^60^ ^]^
MAPbBr_3_	‐	−0.03 V, 906 ms	14.3	^[^ ^61^ ^]^
MAPbBr_3_	0.090 eV	3 V, 10.5 ms	8 × 10^6^	^[^ ^21^ ^]^
FAPbBr_3_	0.090 eV	3 V, 10.5 ms	2.76 × 10^8^	^[^ ^21^ ^]^
CsPbBr_3_	0.090 eV	3 V, 10.5 ms	1.84 × 10^9^	^[^ ^21^ ^]^
MAPbClBr_2_	–	−0.1 V, 25 ms	5.8 × 10^3^	^[^ ^62^ ^]^
PEA_2_PbBr_4_	–	3 V, 10 ms	4 × 10^2^	^[^ ^63^ ^]^
PEA_2_PbBr_4_	–	1.3 V, 100 µs	6.5 × 10^8^	^[^ ^64^ ^]^
(PEA)_2_MA*_n_*_−1_Pb*_n_* Br_3_ *_n_*_+1_	0.160 eV	0.02 V, 100 ms	0.7	^[^ ^48^ ^]^
MA_3_Sb_2_Br_9_	–	0.05 V, 500 µs	9.35 × 10^5^	^[^ ^8^ ^]^
Cs_3_Cu_2_I_5_	0.2 ≈ 0.4 eV	0.1 V, 500 µs	20.55	^[^ ^9^ ^]^
FA‐Bi‐I (2D+0D) mixed phase	0.084 eV	0.02 V, 300 µs	0.58 (7853 µm^2^) 0.061 (1963 µm^2^)	This work

^a^ MA, FA and PEA represent methylammonium, formamidinium and phentylammonium, respectively.

**Figure 5:**
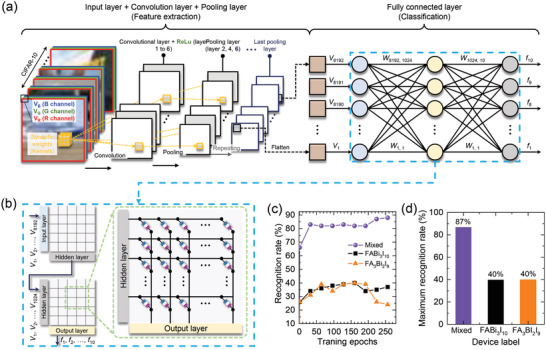
Training and inference tasks for the CIFAR‐10 dataset. a) CIFAR‐10 dataset and a convolutional neural network consisting of the proposed memristor device. b) Conceptual schematic of the neural network containing the memristor devices corresponding to fully connected layer (7^th^ and 8^th^ layers). c) Recognition rate and d) maximum recognition rate of the mixed phase‐, FABi_3_I_10_‐, and FA_3_Bi_2_I_9_‐based memristor devices.

Finally, we have conducted training and inference tasks for the CIFAR‐10 dataset to confirm the feasibility of our memristor‐based synaptic device toward CNN. The “DNN+ NeuroSim”, which is an integrated framework to benchmark compute‐in‐memory (CIM) accelerators for deep neural networks, is used for this work.^[^ ^25^ ^]^ As shown in **Figure** **5**a, the input layer, convolution layers (at from the 1^st^ to 6^th^ layers) and pooling layers (at the 2^nd^, 4^th^, and 6^th^ layers) form the feature extraction part (the left panel of Figure 5a), and the fully connected layers (at the 7^th^ and 8^th^ layers) make the classification part (the right panel of Figure 5a). The CIFAR‐10 dataset consisting of 50 000 training data and 10 000 inference data is applied to the input layer, where the input image pixels and kernels correspond to input voltage signals (*V*) and synaptic weights (*W*), respectively.^[^ ^65^ ^]^ Here, experimentally obtained dynamic range, non‐linearity, number of conductance states, and minimum/maximum conductance values are used as synaptic characteristics for CNN simulation. For training the CIFAR‐10 dataset, two different operations are required: feed‐forward and backpropagation. In the feed‐forward operation, the input voltage signals are convolved by multiple kernels, resulting in the convolved feature maps composed of the current signals (*I* = *V* × *W*). These current signals then pass through the ReLU (ReLU = Rectified linear unit) activation function,^[^ ^66^ ^]^ where this activation process converts the convolved feature maps to the activation feature maps consisting of the voltage signals (*V* = f_ReLU_(*I*)). The activation feature maps are transferred to the next convolution layer (the 2^nd^ layer), and other activation feature maps are generated by repeating the previous convolution process and activation process. After that, the activation feature maps are passed to the pooling layer, where the feature maps are reduced and highlighted via max‐pooling operation. These pooled feature maps are passed to the next convolution layer (the 3^rd^ layer), converted into voltage signals through the ReLU activation function as before, and transferred to the 4^th^ convolution layer. After completing the feature extraction by repeating the process from the 1^st^ layer to the 3^rd^ layer once more, the pooled feature maps consisting of various features of the input image are extracted from the last pooling layer (the 6^th^ layer) and these feature signals are flattened into a 1 × 8912 array for the classification process. The fully connected layers consist of input/hidden/output neuron layers and synapse layers (the 7^th^ and 8^th^ layers) in the classification part (Figure 5b). The flattened signals (*V*) go through the 7^th^ synapse layer, converting to current signals. These current signals are then transformed to voltage signals via the ReLU activation function in the hidden neuron layer. Finally, the voltage signals from the hidden layer pass through the last synapse layer (the 8^th^ layer) and the output neuron. In the backpropagation operation,^[^ ^67^ ^]^ is calculated by the difference between the label values (*K*) and the output signals (*V*_O_). The synaptic weights, which exist in the feature extraction process (kernels) and classification process (7^th^ and 8^th^ synapse layers), are updated from the last output layer to the first convolution layer.

After the training task for 50 000 CIFAR‐10 training images, we compare the recognition rates for the mixed phase with pure 2D and 0D materials via the inference simulation with 10 000 inference images. As shown in Figure 5c, the recognition rate of the network using the mixed phase synapses is remarkably different from the other cases. Figure 5d shows the extracted maximum recognition rates of the networks based on the mixed phase‐, 2D FABi_3_I_10_‐, and 0D FA_3_Bi_2_I_9_‐based synaptic devices. The maximum recognition rate for the mixed phase is approximately 87%, which is significantly higher than the other two cases (approximately 40%). This result is attributed to the P/D characteristics such as dynamic range and asymmetricity,^[^ ^2^, ^65^ ^]^ As aforementioned, the high dynamic range and low asymmetricity for the mixed phase‐based synapse contribute to a high recognition rate, while relatively low recognition rates for pure 2D FABi_3_I_10_ and 0D FA_3_Bi_2_I_9_ are due to insufficient dynamic range and high asymmetricity. We have additionally fabricated 3 memristor devices employing the mixed phase to investigate synaptic behavior more in detail (Table S5). Exploiting values in Table S5, Supporting Information, we conducted training and inference tasks for the CIFAR‐10 dataset to compare recognition rates.^[^ ^4^ ^]^ As shown in Figure S15, Supporting Information, our synaptic devices show a maximum recognition rate of 87% and low device‐to‐device variation with relative standard deviation below 1%.^[^ ^4^ ^]^ Moreover, we confirm the excellence in recognition rate for the mixed phase‐based synapses via training and inference tasks for the modified National Institute of Standards and Technology (MNIST) patterns using the NeuroSim+ MLP simulator (Figure S16, Supporting Information).^[^ ^26^ ^]^

## 3 Conclusion

In case of general oxides and halide perovskite materials, defects for large dynamic range and good linearity act as dopant, which leads to an increase in overall current and thereby an increase in energy consumption. Thus, novel materials are required to fulfill low energy consumption in spite of high defect density. We developed the 2D‐0D mixed phase based on organic bismuth iodide enabling large dynamic range, superior linearity and low energy consumption for highly efficient neuromorphic computing. As compared to highly crystalline 2D FABi_3_I_10_ and 0D FA_3_Bi_2_I_9_, low crystallinity due to phase miscibility was found to be beneficial to lowering activation energy for migration of iodide vacancies. Despite low activation energy and high carrier density, conductivity of the mixed phase was much lower than those of the pure 2D and 0D phases because of the is‐situ formed Type‐I band structure. Thus, the developed mixed phase with low crystallinity was found to be beneficial to not only energy consumption and scalability due to low conductivity but also linearity and dynamic range due to large defect density and low activation energy for migration of iodide vacancies, which eventually realized the recognition rate accuracy of 87% in CNNs. Our unique design of switching materials suggests direction for manufacturing memristor devices to realize characteristic for neuromorphic computing.

## Conflict of Interest

The authors declare no conflict of interest.

## Supporting information

Supporting Information

## Data Availability

The data that support the findings of this study are available in the supplementary material of this article.
