# Supplementary material for: Mixed‐Dimensional Formamidinium Bismuth Iodides Featuring In‐Situ Formed Type‐I Band Structure for Convolution Neural Networks
Source: Adv Sci (Weinh). 2022 Mar 20;9(14):2200168. doi: 10.1002/advs.202200168 (PMC9108665; doi:10.1002/advs.202200168)
Supplement: Supplementary file 1 — Supporting Information [file ADVS-9-2200168-s001.pdf]

Supporting Information for

Mixed-dimensional Formamidinium Bismuth Iodides Featuring in-situ Formed Type-I Band Structure for Convolution Neural Networks

June-Mo Yang[‡a], Ju-Hee Lee[‡b], Young-Kwang Jung[d], So-Yeon Kim[a], Jeong-Hoon Kim[b], Seul-Gi Kim[a], Jeong-Hyeon Kim[a], Seunghwan Seo[b], Dong-Am Park[a], Jin-Wook Lee[c], Aron Walsh[d,e], Jin-Hong Park[b,c]* and Nam-Gyu Park[a]*.

[a]School of Chemical Engineering, Energy Frontier Laboratory, Sungkyunkwan University, Suwon 16419, Korea.

[b]Department of Electrical and Computer Engineering, Sungkyunkwan University, Suwon 16419, Korea

[c]Sungkyunkwan Advanced Institute of Nanotechnology (SAINT), Sungkyunkwan University, Suwon 16419, Korea

[d]Department of Materials Science and Engineering, Yonsei University, Seoul 03722, Korea

[e]Department of Materials, Imperial College London, London SW7 2AZ, U.K.

[‡]These authors contributed equally to this work.

*Corresponding author

J. -H. P.: E-mail: jhpark9@skku.edu

N.-G. P.: E-mail: npark@skku.edu

Experimental Section

Synthesis of Formamidinium Iodide. Formamidinium iodide (FAI = $HC(NH_2)_2I$) was synthesized by reacting 20 g of formamidine acetate salt ($NH = CHNH_2{\cdot}CH_3COOH$, Aldrich, 99%) with 30 mL of HI (Aldrich 57 wt% in H_2O). HI was added to formamidine acetate salt in round-bottom flask and stirred with magnetic bar for 2 h. The brown precipitate was collected by rotary evaporator at 65 °C, washed with diethyl ether four times, and

recrystallized with ethanol to get white powder. The white powder was dried for 24 h in vacuum oven.

Fabrication of devices. ITO-coated substrates were cleaned with detergent, ethanol, acetone and ethanol by ultrasonication, which was followed by O_2 plasma treatment for 10 min or Ultraviolet-ozone (UVO) for 1 h. The PEDOT:PSS (Al 4083, Heraeus) solution of 20 μl (describe in detail how to prepare PEDOT:PSS solution) was deposited on the ITO-coated substrates (1.5 cm x 1.5 cm) by spin-coating method at 4,000 rpm for 30 s (acceleration = 1200 rpm^{-1}), which was annealed at 150 ^oC for 15min. Formamidinium bismuth iodides precursor solutions were prepared by dissolving 1 mmol of BiI_3 (589 mg) (Aldrich 99.0%) and x mol of formamidinium iodide (FAI) (x = 0.33 mmol (57.2 mg) for $FABi_3I_{10}$, 0.66 mmol (114 mg) for the mixed phase and 1.5 mmol (258 mg) for $FA_3Bi_2I_9$ and) in 1.2 ml of DMF (Sigma-Aldrich 99.8%). Describe how to prepare FAI here.(I added previous paragraph) The precursor solution was spin-coated on the PEDOT:PSS-coated ITO substrate at 4,000 rpm (acceleration = 1200 rpm^{-1}) for 20 s, where 0.2 ml of toluene (Aldrich, 99.8%) was dripped while rotating (15 s after spinning). The spin-coated film was annealed at 100 ^oC for 10 min For the top electrode, a shadow mask with holes of 200, 100 and 50 μm in diameter was used, where 150 nm-thick Ag top electrode was thermally evaporated on the fromamidinium bismuth iodide films under 1.0×10^{-6} Torr at room temperature.

Electrical characterization. I-V curves, analog resistive switching and SCLC were measured by Keithley 4200 semiconductor parametric analyzer. All electrical measurements were performed in vacuum in a probe station (MS Tech) under 10^{-2} Torr to minimize the influence of the moisture and oxygen in air on device performance.

Measurements for characterization of materials. X-ray diffraction (XRD) data were

obtained by D8 ADVANCE diffractometer (Bruker, 18 kW) with Cu Kα radiation (λ = 1.5406 Å). Scanning electron microscope (SEM) images were measured by SEM (JSM7000F, JEOL).

Capacitance measurement. Devices with ITO/(FA-Bi-I)/Au structure were prepared for impedance spectroscopy (IS) measurements. Capacitance was measured using an impedance spectroscopy (IS) measurements (PGSTAT 128N (Autolab, Eco-Chemie)) with small perturbation of AC 20 mV at frequencies ranging from 100 mHz to 1MHz in the dark.

First-principles calculations. The underlying DFT calculations were performed using the Vienna Ab initio Simulation Package (VASP).[1,2] Projector augmented-wave (PAW) [3,4] pseudopotentials were employed to treat core atomic states where the valence electron configurations of Cs, Bi, and I are explicitly considered as $5s^2 5p6^6 s^1$, $5d^{10} 6s^2 6p^3$, and $5s^2 5p^5$, respectively. For all calculations the Perdew–Burke–Ernzerhof exchange-correlation functional revised for solids (PBEsol) [5] was used. A plane-wave kinetic cutoff energy was set to 700 eV. Convergence criterion of the total energy was set to 10−6 eV and of forces on each atoms was set to 10−2 eV/Å. To model the 0D/2D interface systems, we constructed superlattice model along c-axis (~50 Å). $Cs_3Bi_2I_9$ and $CsBi_3I_{10}$ were chosen as model compositions to avoid issues arising from orientations and disorder of molecular cations in the model super lattice. The Brillouin-zone integrations were performed with a Γ-centered k-point grid of 6 × 6 × 2 for the primitive $Cs_3Bi_2I_9$ and $CsBi_3I_{10}$ unit cells and of 6 × 6 × 1 for the interface super lattice. The initial atomic structure of $CsBi_3I_{10}$ was adopted from Ref. 6. During the structural optimization of the super lattice, we relaxed lattice vectors and atomic coordinates with a constraint of cell geometry in a hexagonal shape. Band offset between $Cs_3Bi_2I_9$ and $CsBi_3I_{10}$ was calculated considering core level shift of Bi 1s states following the procedure outlined in Ref. 7.

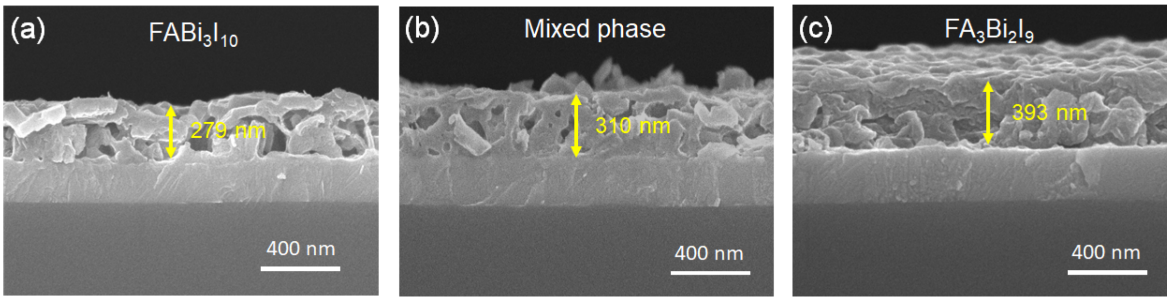

Figure S1. Cross sectional SEM images related to measurement of (a) $FABi_3I_{10}$, (b) the mixed phase and (c) $FA_3Bi_2I_9$.

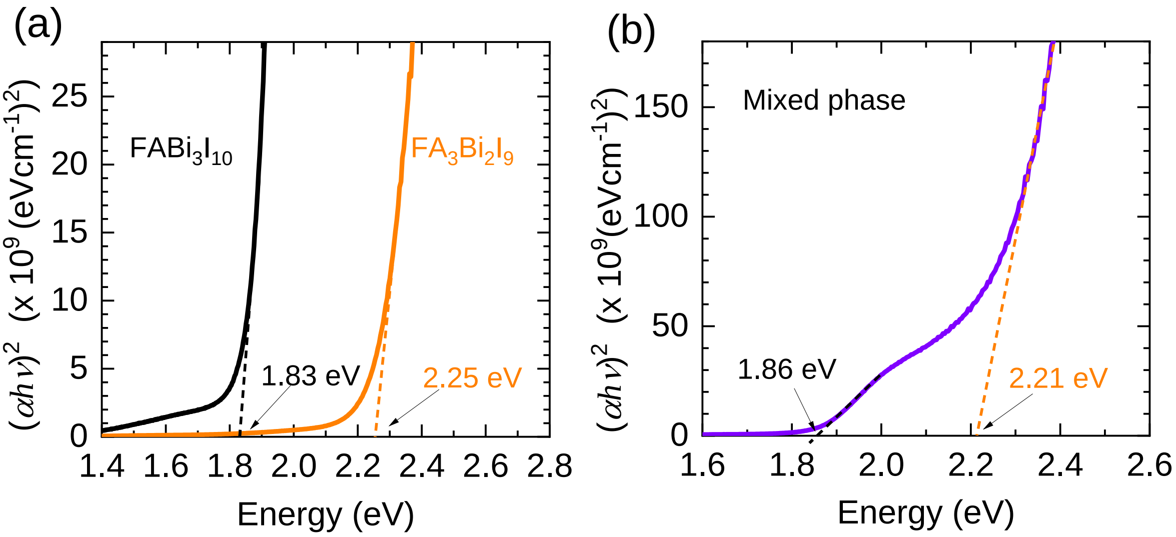

Figure S2. Tauc plots of (a) $FABi_3I_{10}$ and $FA_3Bi_2I_9$ and (b) the mixed phase.

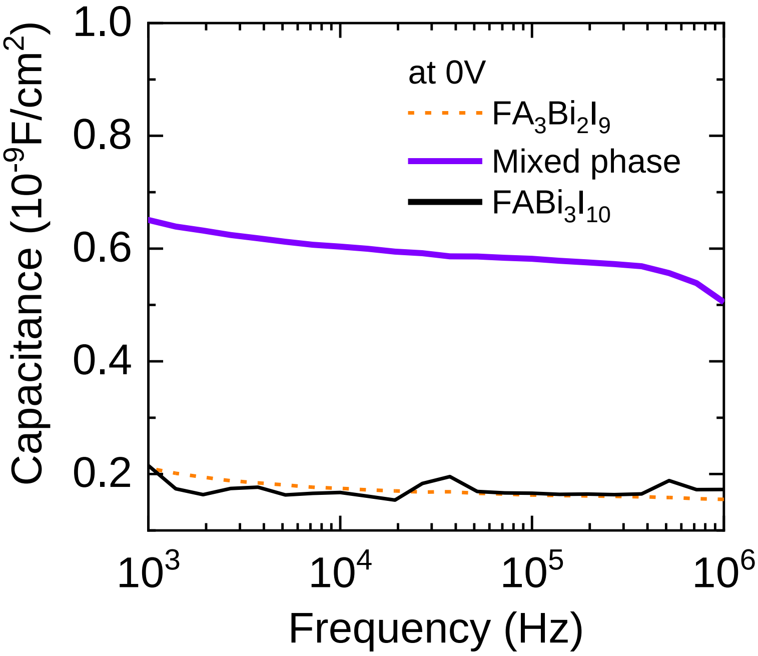

Figure S3. Capacitance–Frequency characteristic of dielectric region for $FABi_3I_{10}$, the mixed phase and $FA_3Bi_2I_9$ using an Au/(FA-Bi-I)/ITO device structure.

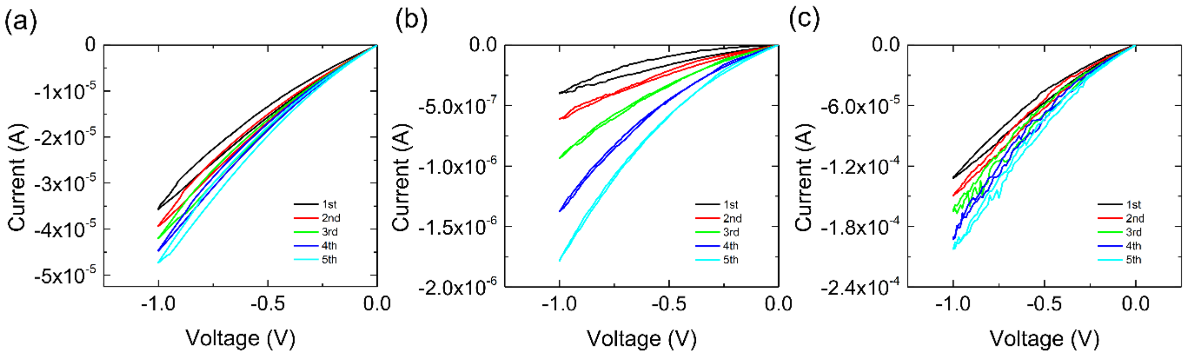

Figure S4. I-V curves for (a) $FABi_3I_{10}$, (b) the mixed phase and (c) $FA_3Bi_2I_9$ when negative voltage sweeps from 0 V to -1 V and then back to 0 V, defined as one cycle, were repeated for five cycles.

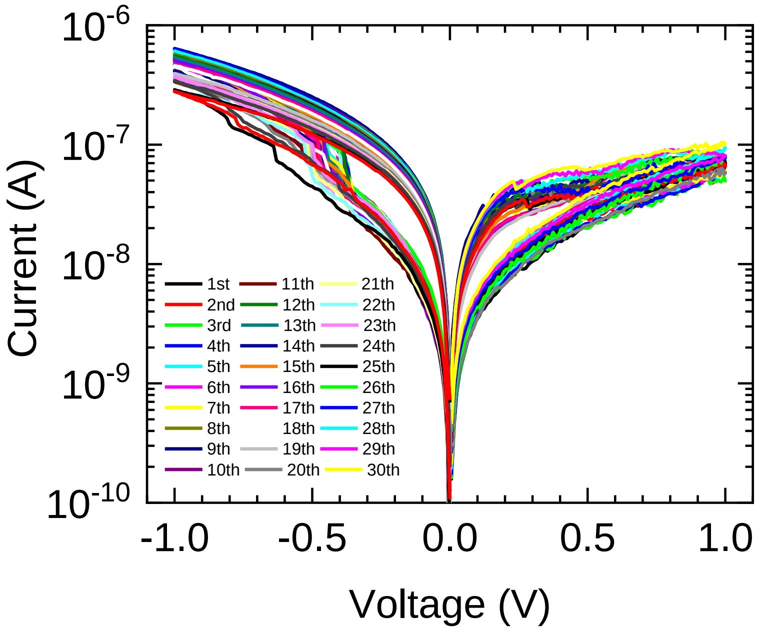

Figure S5. I–V characteristics of repeated switching of the mixed phase-based memristor.

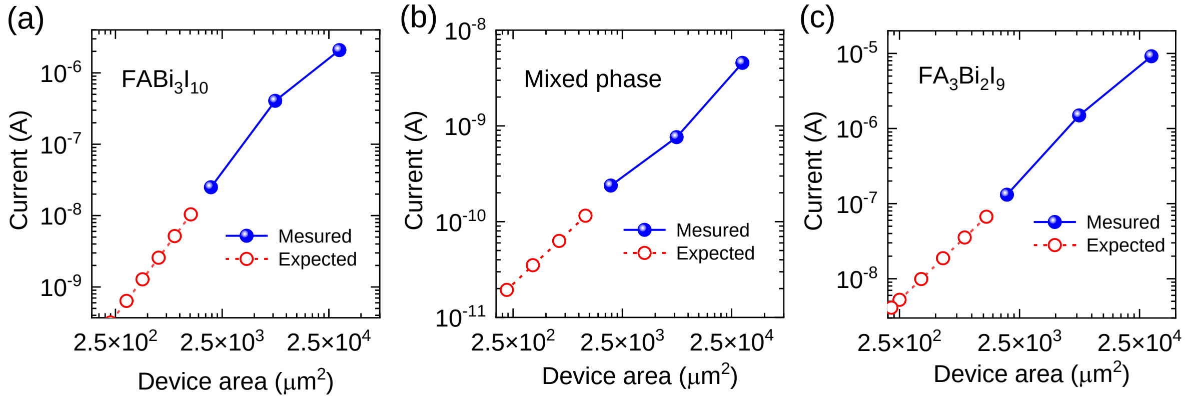

Figure S6. Dependence of current on active area for (a) $FABi_3I_{10}$, (b) the mixed phase and (c) $FA_3Bi_2I_9$, measured using an Ag/(FA-Bi-I)/PEDOT:PSS/ITO device structure. Current was measured at 0.02 V. Expected data were extrapolated from the measured data.

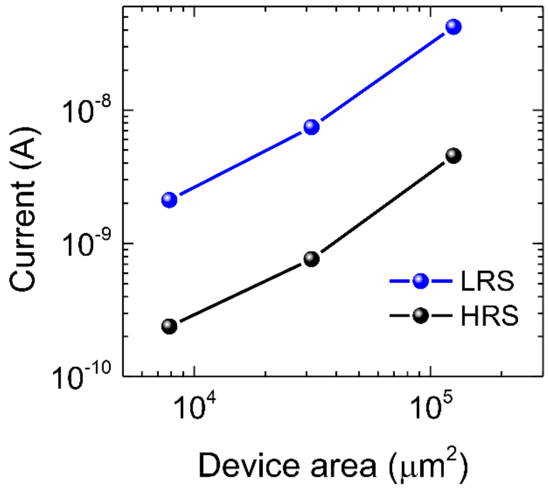

Figure S7. Dependence of current on active area for HRS and LRS of the mixed phase, measured using an Ag/(FA-Bi-I)/PEDOT:PSS/ITO device structure. Current was measured at 0.02 V.

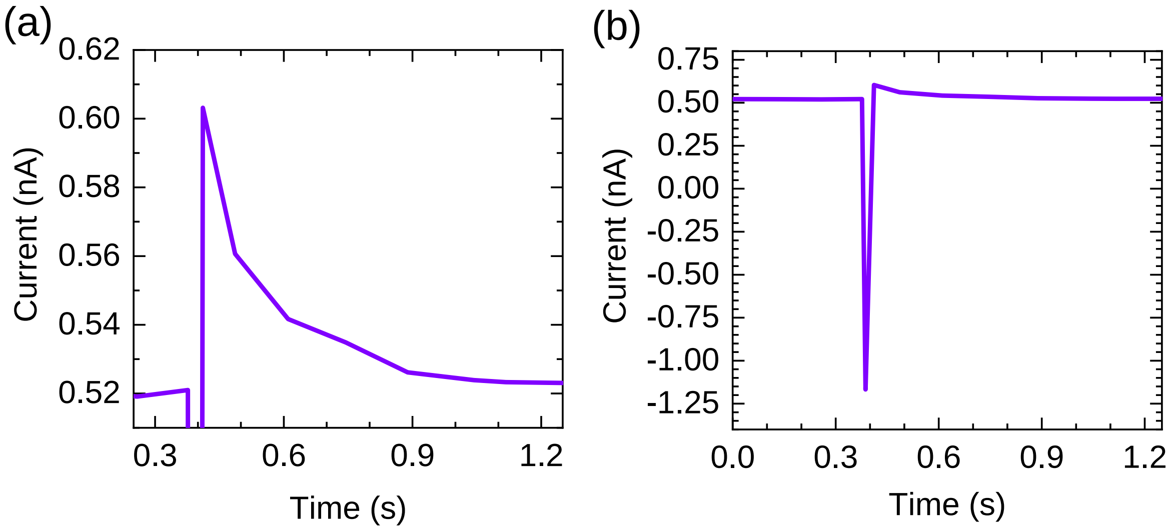

Figure S8. (a) EPSC characteristics and (b) peak current for the 7,856 μm^2-sized mixed phase-based memristor measured at a 25 μs pulse of -0.02 V.

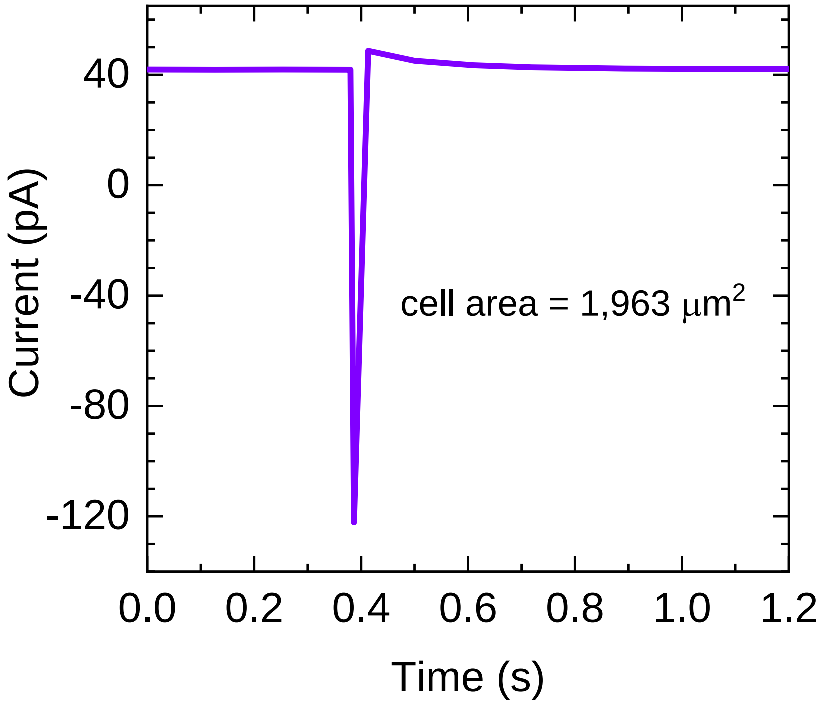

Figure S9. EPSC characteristics with peak current for the 1,963 μm^2-sized mixed phase-based memristor measured at a 25 μs pulse of -0.02 V.

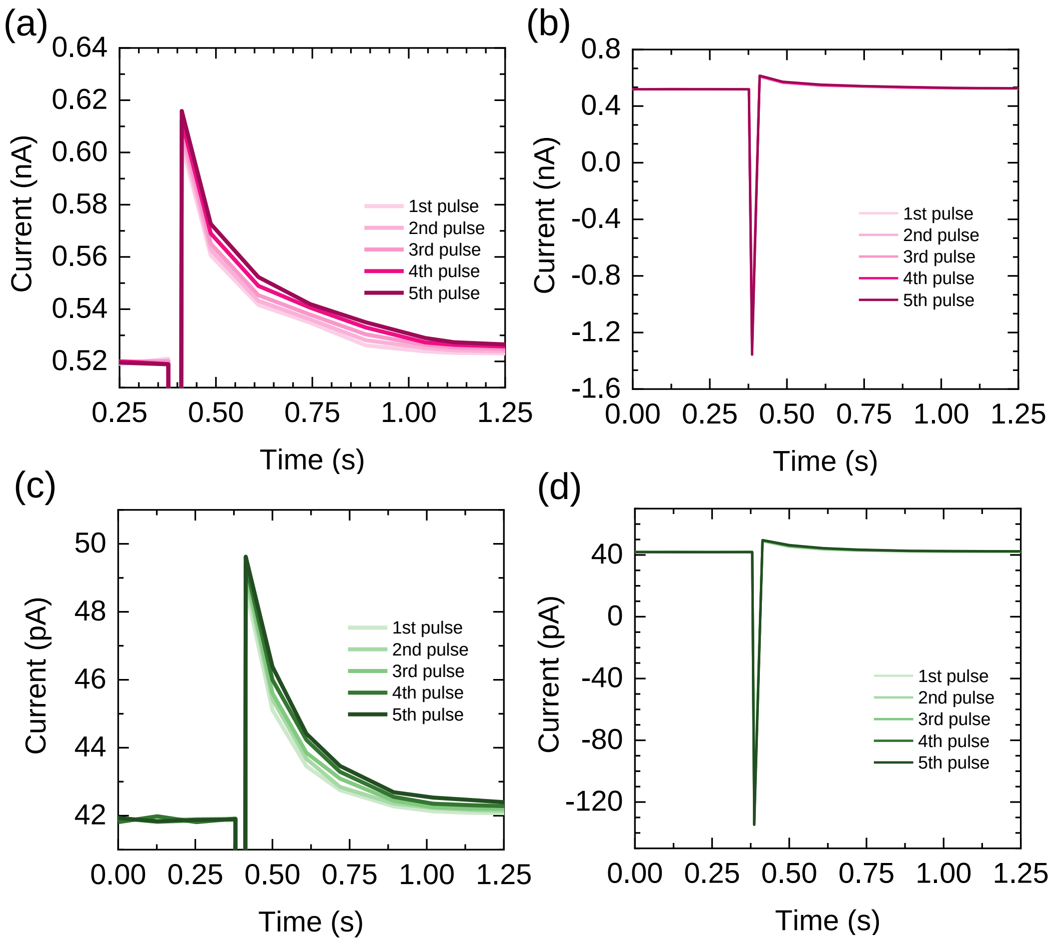

Figure S10. SNDP characteristics measured by applying a 25 μs pulse of -0.02 V for 5 times for of the mixed phase-based memristors with cell area of (a, b) 7,856 μm^2 and (c, d) 1,963 μm^2.

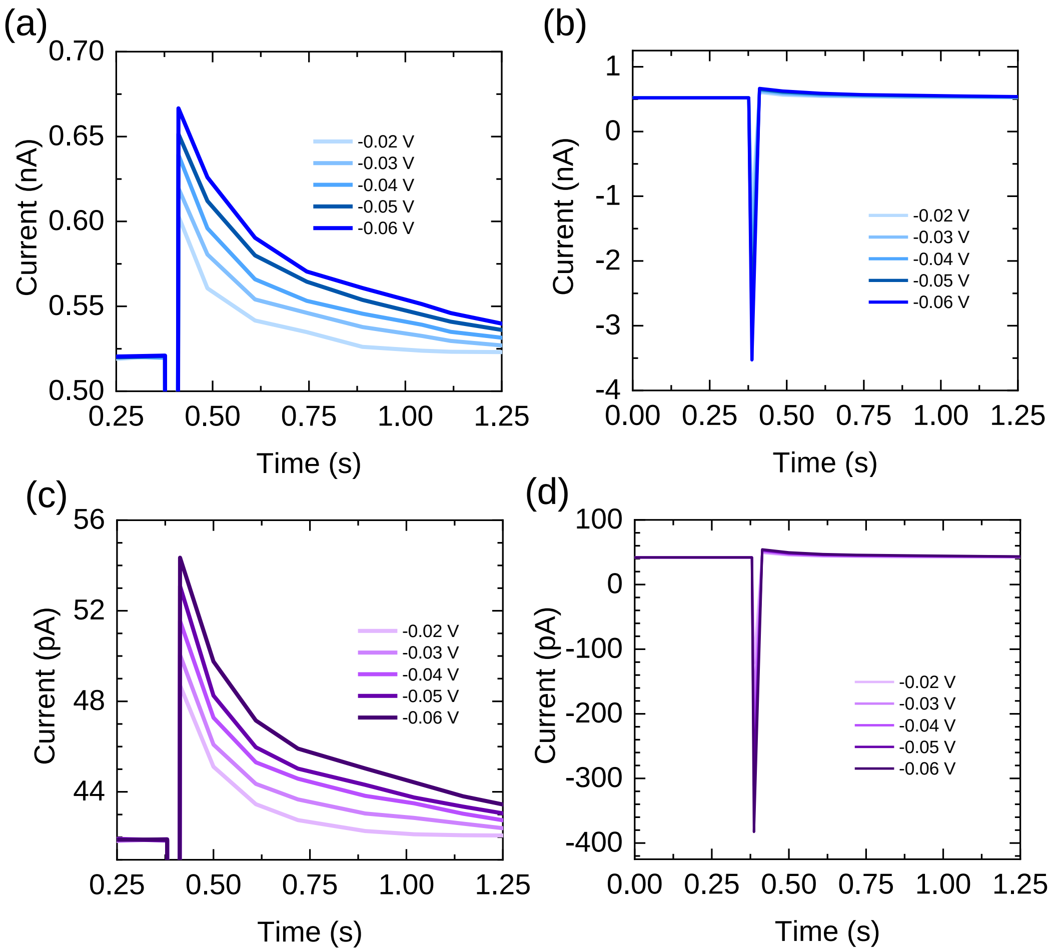

Figure S11. SVDP characteristics measured at 25 μs pulses of 0.1 V, 0.2 V, 0.3 V, 0.4 V and 0.5 V for the mixed phase-based memristors with cell area of (a, b) 7,856 μm^2 and (c, d) 1,963 μm^2.

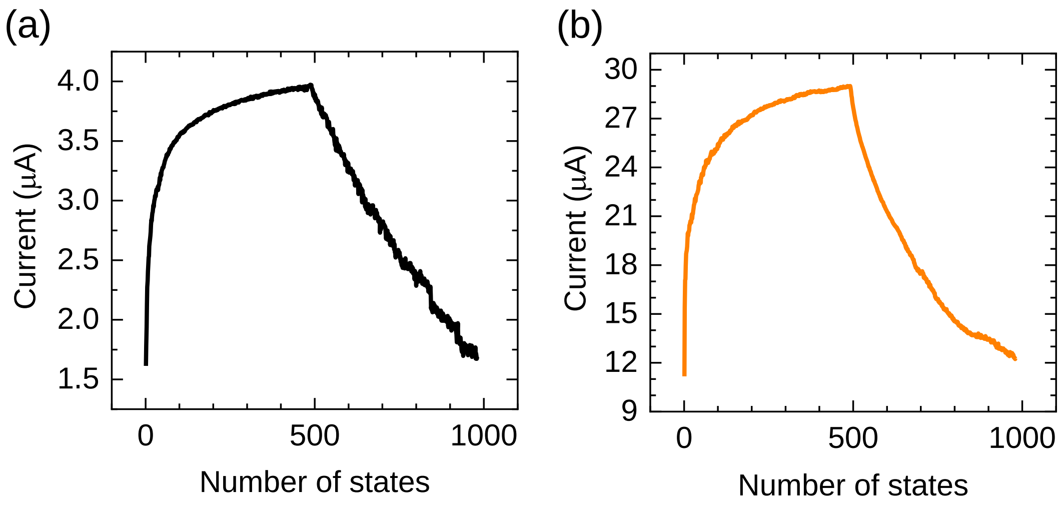

Figure S12. Potentiation and depression for the memristor based on (a) $FABi_3I_{10}$ and (b) $FA_3Bi_2I_9$ depending on the number of states, where 500 consecutive negative pulses (-0.6 V, 300 μs) were applied for potentiation and 500 positive pulses (0.2 V, 300 μs) for depression. 0.02 V reading voltage was applied after each negative and positive pulse.

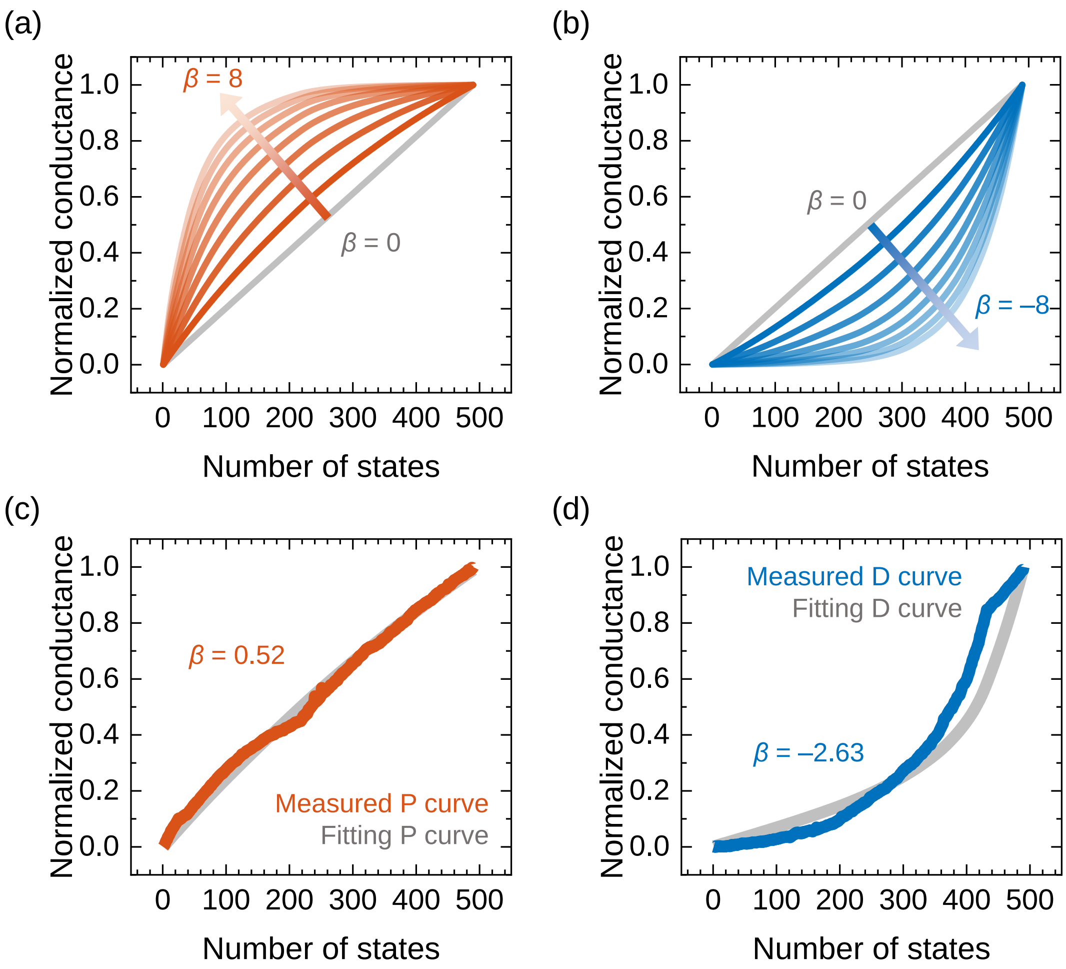

Figure S13. Non-linearity (β) analysis of P/D characteristic curves. (a, b) Normalized conductance (G) curves with respect to β ranging from 0 to 8. Measured and fitting curves in (c) P and (d) D regions, where β = 0.52 and –2.63, respectively.

There are several methods to calculate the non-linearity (β) of the P/D characteristic curve.[8-10] Among them, we chose a method to tune A_P and A_D (see the below equations) for finding the G_P/G_D curves matched well to the measured P/D curves.[11] The G_P/G_D curve model with the number of pulses (P) is represented as follows:

$$G_P = B_P(1 - \exp(-P/A_P)) + G_{min} \quad (1)$$

$$G_D = -B_D(1 - \exp((P - P_{max})/A_D)) + G_{max} \quad (2)$$

$$B_{P,D} = (G_{max} - G_{min})/(1 - \exp(-P_{max}/A_{P,D})) \quad (3)$$

where G_P and G_D are the conductance values for P and D, respectively. G_{max}, G_{min}, and P_{max}

are the measured data representing the maximum conductance, minimum conductance, and maximum pulse number, respectively. $B_{P, D}$ is a fitting constant to normalize the conductance range. A_P and A_D are parameters that determine the nonlinearities of the weight update in the P and D regions that are directly related to the β values.[8-11] The G_P/G_D curves with respect to the β ranging from 0 to 8 are displayed in Figure S11a and b. By adjusting the A_P and A_D values, the G_P/G_D curves were fitted to the measured P/D curves, and accordingly, the β values were determined (Figure S11c and d).

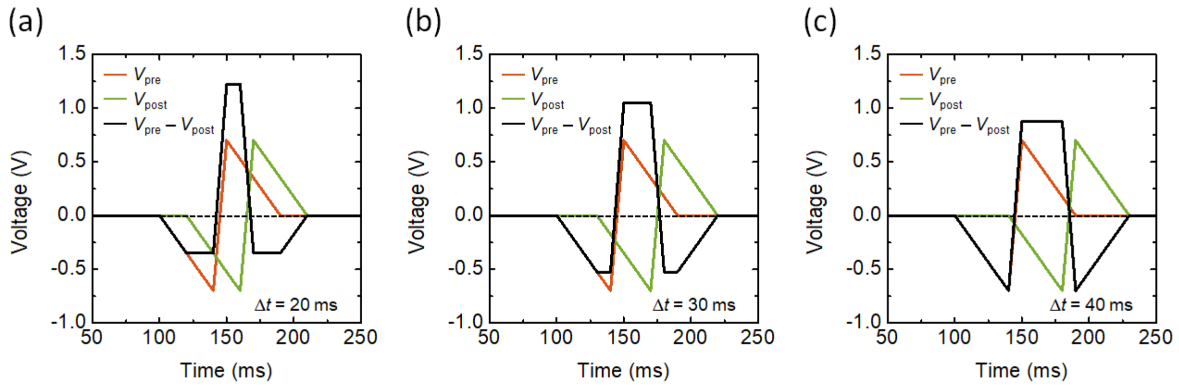

Figure S14. Shapes of $V_{pre} - V_{post}$ as the time interval (Δt) increases

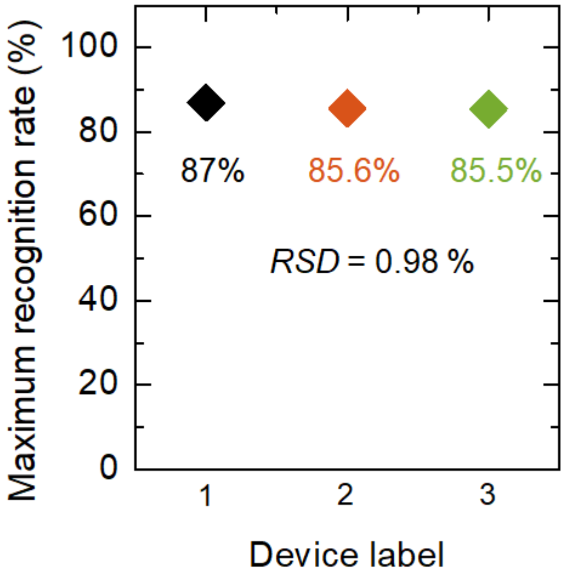

Figure S15. Maximum recognition rates for three mixed phase-based memristor devices

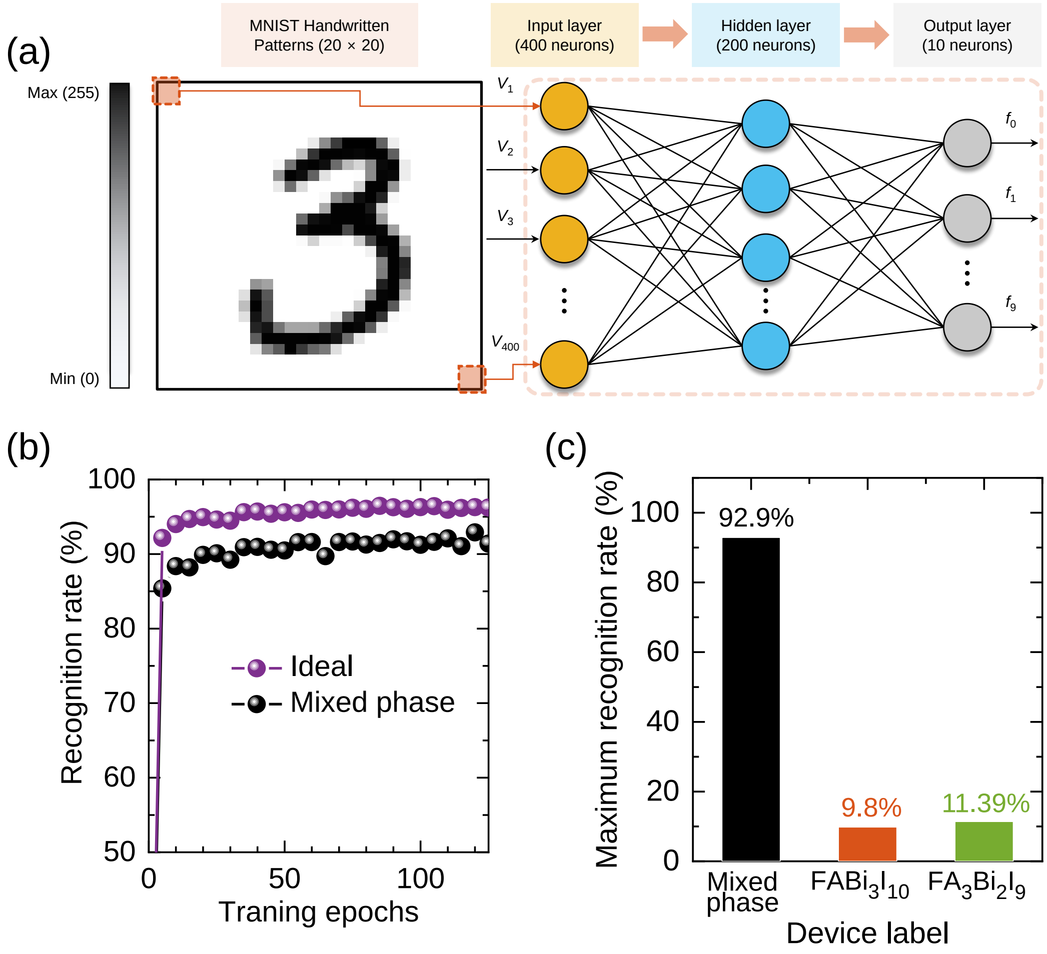

Figure S16. Training and inference tasks for MNIST dataset. (a) MNIST dataset and schematic illustration of multi-layer ANN with a size of 400 × 200 × 10. (b) Comparison of recognition rates of ideal synapse and our memristor device. (c) Maximum recognition rate of the mixed phase-, $FABi_3I_{10}$-, and $FA_3Bi_2I_9$-based memristor devices.

Table S1. Data for calculation of carrier mobility, carrier density and defect density.

	$FABi_3I_{10}$	Mixed phase	$FA_3Bi_2I_9$
A_1	20.68×10^{-6} m^3	19.77×10^{-6} m^3	21.99×10^{-6} m^3
A_2	3.14×10^{-8} m^3	3.14×10^{-8} m^3	3.14×10^{-8} m^3
C (Capacitance)	1.72×10^{-9} F	6.51×10^{-9} F	2.11×10^{-9} F
e (Elementary charge)	1.602×10^{-19} C	1.602×10^{-19} C	1.602×10^{-19} C
L (Film thickness)	279×10^{-9} m	310×10^{-9} m	339×10^{-9} m
$\frac{I}{V^2}$ in SCLC region	8.24×10^{-6} A/V^2	6.82×10^{-9} A/V^2	2.71×10^{-4} A/V^2
$\frac{I}{V}$ in ohmic region	2.68×10^{-6}A/V	1.77×10^{-9} A/V	2.04×10^{-4} A/V
V_{TFL}	0.64 V	1.17 V	0.98 V

Table S2. Electrical properties calculated by using data in Table S1.

	$FABi_3I_{10}$	Mixed phase	$FA_3Bi_2I_9$
Mobility	2.18×10^{-3} cm^2 $V^{-1}s^{-1}$	5.47×10^{-7} cm^2 $V^{-1}s^{-1}$	9.19×10^{-2} cm^2 $V^{-1}s^{-1}$
Carrier density	6.82×10^{14} cm^{-3}	1.99×10^{15} cm^{-3}	1.50×10^{15} cm^{-3}
Defect density	2.38×10^{15} cm^{-3}	1.55×10^{16} cm^{-3}	3.46×10^{15} cm^{-3}
FWHM	0.17	0.25 and 0.26	0.18
Crystal size	46.7 nm	31.8 nm and 30.5 nm	44.1 nm
Conductivity	2.38×10^{-7} S cm^{-1}	1.75×10^{-10} S cm^{-1}	2.21×10^{-5} S cm^{-1}

Table S3. Current and energy consumption required to conduct SNDP for the mixed phase.

Cell area		**1st pulse**	**2nd pulse**	**3rd pulse**	**4th pulse**	**5th pulse**
1,963 μm^2	ΔG	0.468157	0.603315	0.767845	0.961269	1.209377
	Peak current	-122.164 pA	-125.164 pA	-127.693 pA	-130.471 pA	-134.768 pA
	Energy consumption	61.08 aJ	62.58 aJ	63.85 aJ	65.24 aJ	67.38 aJ
7,853 μm^2	ΔG	0.589737	0.739551	0.91802	1.095913	1.245535
	Peak current	-1.16694 nA	-1.20361 nA	-1.25194 nA	-1.30427 nA	-1.35691 nA
	Energy consumption	583.47 aJ	601.81 aJ	625.97 aJ	652.14 aJ	678.46 aJ

Table S4. Current and energy consumption required to conduct SVDP for the mixed phase.

Cell area		**Bias V -0.02 V**	**Bias V -0.03 V**	**Bias V -0.04 V**	**Bias V -0.05 V**	**Bias V -0.06 V**
1,963 μm^2	ΔG	0.468157304	1.106774525	1.883747634	2.623173113	3.484967724
	Peak current	-122.164 pA	-189.613 pA	-251.416 pA	-310.848 pA	-382.823 pA
	Energy consumption	61.08 aJ	94.80 aJ	125.71 aJ	174.07 aJ	191.41 aJ
7,853 μm^2	ΔG	0.500491	1.248825	2.147145	2.895422	3.630964
	Peak current	-1.16694 nA	-1.75364 nA	-2.32777 nA	-3.01904 nA	-3.52918 nA
	Energy consumption	583.47 aJ	876.82 aJ	1.163 fJ	1.510 fJ	1.765 fJ

Table S5. Synaptic characteristics for training/recognition tasks

Sample #	#1	#2	#3
Dynamic range	14.99	15.2	14.41
Asymmetricity (Non-linearity (P/D))	3.15 (0.52/–2.63)	3.47 (0.49/–2.98)	3.47 (0.57/–2.9)
# of conductance P/D states	490/490	459/546	489/550
Recognition rate	87%	85.6%	85.5%

References

[1] G. Kresse, J. Furthmüller. Phys. Rev. B: Condens. Matter Mater. Phys. **1996**, 54, 11169–11186.

[2] G. Kresse, J. Furthmüller. Comput. Mater. Sci. **1996**, 6, 15–50.

[3] G. Kresse, D. Joubert, Phys. Rev. B: Condens. Matter Mater. Phys. **1999**, 59, 1758–1775

[4] P. E. Blöchl, Phys. Rev. B: Condens. Matter Mater. Phys. **1994**, 50, 17953–17979.

[5] J. P. Perdew, A. Ruzsinszky, G. I. Csonka, O. A. Vydrov, G. E. Scuseria, L. A. Constantin, X. Zhou, and K. Burke. Phys. Rev. Lett. **2008**, 100, 136406.

[6] J. Shin, M. Kim, S. Jung, C. S. Kim, J. Park, A. Song, K.-B. Chung, S.-H. Jin, J. H. Lee, M. Song. Nano Research. **2018**, 11, 6283–6293

[7] K. T. Butler, J. M. Frost, A. Walsh. Mater. Horiz. **2015**, 2, 228–231.

[8] P.-Y. Chen, X. Peng, S. Yu, in 2017 IEEE Int. Electron Devices Meet., IEEE, 2017, pp. 6.1.1-6.1.4.

[9] C. Sen Yang, D. S. Shang, N. Liu, E. J. Fuller, S. Agrawal, A. A. Talin, Y. Q. Li, B. G. Shen, Y. Sun, Adv. Funct. Mater. 2018, 28, 1.

[10] L. Yin, C. Han, Q. Zhang, Z. Ni, S. Zhao, K. Wang, D. Li, M. Xu, H. Wu, X. Pi, D. Yang, Nano Energy 2019, 63, 103859.

[11] S. Gandla, M. Naqi, M. Lee, J. J. Lee, Y. Won, P. Pujar, J. Kim, S. Lee, S. Kim, Adv. Mater. Technol. 2020, 5, 1.
